# Impaired threat prioritisation after selective bilateral amygdala lesions

**DOI:** 10.1016/j.cortex.2014.08.017

**Published:** 2015-02

**Authors:** Dominik R. Bach, Rene Hurlemann, Raymond J. Dolan

**Affiliations:** a Wellcome Trust Centre for Neuroimaging, University College London, UK; b Department of Psychiatry, Psychotherapy, and Psychosomatics, University of Zurich, Switzerland; c Department of Psychiatry, University of Bonn, Germany

**Keywords:** Amygdala lesion, Threat, Fear, Urbach–Wiethe, Facial expression, Serial search

## Abstract

The amygdala is proposed to process threat-related information in non-human animals. In humans, empirical evidence from lesion studies has provided the strongest evidence for a role in emotional face recognition and social judgement. Here we use a face-in-the-crowd (FITC) task which in healthy control individuals reveals prioritised threat processing, evident in faster serial search for angry compared to happy target faces. We investigate AM and BG, two individuals with bilateral amygdala lesions due to Urbach–Wiethe syndrome, and 16 control individuals. In lesion patients we show a reversal of a threat detection advantage indicating a profound impairment in prioritising threat information. This is the first direct demonstration that human amygdala lesions impair prioritisation of threatening faces, providing evidence that this structure has a causal role in responding to imminent danger.

## 1 Introduction

Extant theories implicate the amygdala in detection and prioritisation of threat-related information (LeDoux, 2000) and hence place it centre stage for disorders from the anxiety and fear spectrum. This view is based primarily on the non-human amygdala's role in learning to predict acute threat, exemplified by fear conditioning. Yet, although several human individuals with selective amygdala lesion (SM, AM, BG) are reported to be impaired in verbal recognition and intensity rating of fearful face expression when there are no time constraints (Adolphs, Tranel, Damasio, & Damasio, 1994; Becker et al., 2012), there is a spared ability in one of these individuals, SM, to detect fearful faces under time constraints, or when no explicit evaluation of the depicted emotion is required (Tsuchiya, Moradi, Felsen, Yamazaki, & Adolphs, 2009). These findings are interpreted as suggesting the human amygdala is not essential for early stages of fear processing but, instead, for modulation of recognition and social judgement (Tsuchiya et al., 2009).

These conflicting views can be reconciled if one assumes that fearful faces – used in previous human lesion studies – are reformulated as representing threat, but not necessarily a threat to the observer. Hence, they constitute an important cue for social communication but not an unambiguous threat signal. A non-human literature posits a role for the amygdala in detection of threat to oneself, rather than to others. In this framework, probing detection of fearful faces does not address the question of threat detection. Angry face expression on the other hand is a more unambiguous threat signal. Yet, the detection of angry facial expression after human amygdala lesion has only been probed in social judgement tasks requiring explicit intensity rating (Adolphs et al., 1994), free verbal response (Becker et al., 2012), or explicit comparison of threat potential (Tsuchiya et al., 2009). Hence, in the present study, we sought to address prioritised processing of angry faces in a task that does not require explicit evaluation.

In healthy humans, angry faces enjoy prioritised processing compared to other face expressions (Bar-Haim, Lamy, Pergamin, Bakermans-Kranenburg, & van IJzendoorn, 2007). Prioritised processing is evident as preferential spatial attention for angry face expression in a dot probe task (Macleod & Mathews, 1988; Macleod, Mathews, & Tata, 1986), as privileged access to memory when capacity is limited in the attentional blink task (de Jong & Martens, 2007), and as quicker response times (RTs) for angry than for happy faces in the face-in-the-crowd (FITC) task (Hampton, Purcell, Bersine, Hansen, & Hansen, 1989; Hansen & Hansen, 1988). Although these early FITC experiments were criticised for use of problematic stimuli (Purcell, Stewart, & Skov, 1996), several subsequent studies revealed similar effects both with photographic (Gilboa-Schechtman, Foa, & Amir, 1999; Horstmann & Bauland, 2006; Williams, Moss, Bradshaw, & Mattingley, 2005) and schematic stimuli (Esteves, 1999; Fox et al., 2000; Horstmann, 2007; Lundqvist & Ohmann, 2005; Ohman, Lundqvist, & Esteves, 2001; Schubo, Gendolla, Meinecke, & Abele, 2006; Tipples, Atkinson, & Young, 2002). Also, when RT is limited, search for angry faces is more precise than for happy faces (Schmidt-Daffy, 2011). In an FITC task, search speed depends linearly on the size of the crowd and is about half as fast when the target is absent than when present (Horstmann & Bauland, 2006). This indicates exhaustive serial search, i.e., each face in the crowd is searched one after the other until either the deviating face is found (which occurs, on average, after searching half of the crowd), or until the entire crowd has been searched and the target found to be absent. Crucially, search slopes are shallower for angry than for happy faces, indicating prioritised processing of threat information and causing more rapid detection of threat than of other stimuli.

Here we used the FITC task to probe prioritisation of angry faces in twin sisters AM and BG, two individuals with relatively selective bilateral amygdala lesions due to congenital Urbach–Wiethe disease (lipoid proteinosis). This disorder often leads to specific calcification of the amygdala that is thought to encroach on this structure gradually over the course of childhood and adolescence (Newton, Rosenberg, Lampert, & O'Brien, 1971). While BG suffered a single epileptic grand-mal seizure aged 12 leading to her diagnosis, AM never had epileptic seizures. Both twins attended regular neurological consultations after this diagnosis, and were recruited for neuropsychological experiments at the age of 21 (Strange, Hurlemann, & Dolan, 2003). Neuropsychological assessment one year before the present study, at the age of 34, (Talmi, Hurlemann, Patin, & Dolan, 2010) revealed impairments in phonemic fluency (Aschenbrenner, Tucha, & Lange, 2000) and short-term concentration (Brickenkamp, 1995). AM but not BG was impaired in figural learning and memory, as shown in the Complex Figure Test (Osterrieth, 1944) and the DCS (Weidlich & Lamberti, 2001). In behavioural experiments, BG was impaired in free verbal recognition of fearful faces, and in startle potentiation by threat-related scenes, and had a reduced social network compared to control participants, while all these functions were intact in AM (Becker et al., 2012). Further, both twins showed reduced anterograde and retrograde interference of emotional pictures on memory (Hurlemann et al., 2007).

On the other hand, the aforementioned neuropsychological assessment (Talmi et al., 2010) revealed average intelligence (L-P-S Leistungsprüfsystem) (Horn, 1983) and intact verbal learning and memory (Rey Auditory Verbal Learning test) (Helmstedter, Lendt, & Lux, 1981) as well as executive function measured with the Trail Making Test (Reitan, 1955), Wisconsin Card Sorting Test (Kongs, Thompson, Iversion, & Heaton, 2000), Stroop test (Bäumler, 1985), and semantic fluency (Aschenbrenner et al., 2000). The twins show neither depression nor anxiety (Hamilton, 1959; 1960). Further, both twins were unimpaired in rapid detection of negative-arousing words (Bach, Talmi, Hurlemann, Patin, & Dolan, 2011), forced-choice recognition of emotional expression in prosody (Bach, Hurlemann, & Dolan, 2013), and framing effects on economic gambles (Talmi et al., 2010).

Given the amygdala damage in AM and BG, and the posited function of the amygdala in prioritising threat information, we hypothesised a reduced angry face advantage in the FITC task in AM and BG, compared to healthy individuals.

## 2 Materials and methods

### 2.1 Design

The task followed a 3 (set size: 1/6/12 items) × 2 (target emotion: angry/happy) × 2 (target absent/present) factorial design with RT as dependent variable. Some previous studies have only analysed slopes of a serial search model. Here, because we did not know whether Urbach–Wiethe patients use a serial search strategy, we analyse both raw RTs and search slopes as dependent variables.

### 2.2 Participants

AM (previously also labelled patient 1) and BG (patient 2) (Becker et al., 2012), aged 35 years at the time of the present experiment, are monozygous twins with congenital Urbach–Wiethe syndrome due to a de novo mutation (Becker et al., 2012). The calcified volumes on high-resolution computer assisted tomography images included the whole basolateral amygdala and most other amygdala nuclei, only sparing anterior amygdaloid and ventral cortical amygdaloid parts at an anterior level, as well as lateral and medial parts of the central amygdaloid nucleus and the amygdalo-hippocampal area at posterior levels. Control participants were included if they were females between the age of 29 and 41 years, and the final sample comprised 16 healthy females with an age of 33.6 ± 3.4 years. They also served as control group for experiment 2 of a previous report (Bach et al., 2013). All participants gave written informed consent, and the study was approved by local ethics committees.

### 2.3 Task & procedure

**Fig. 1:**
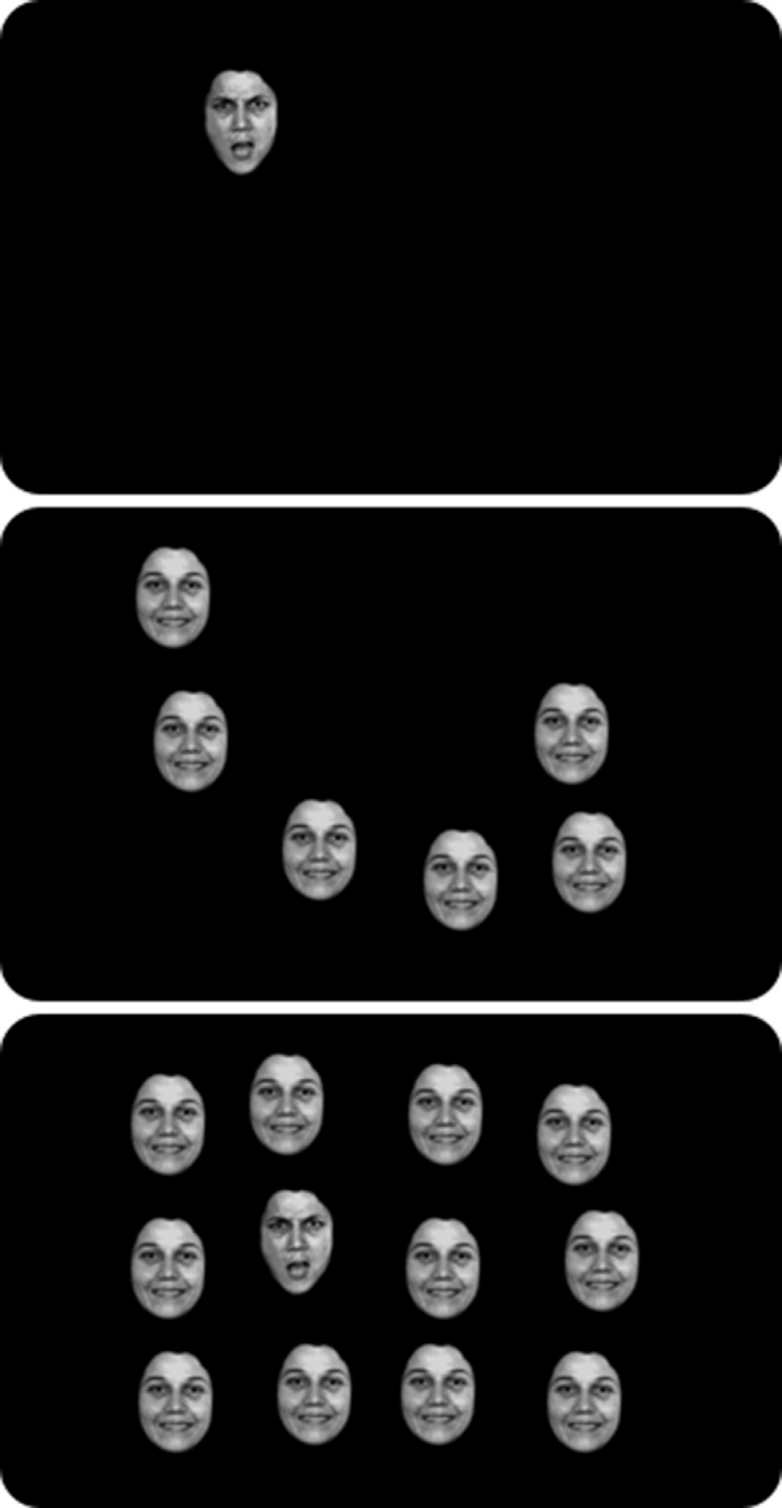
Schematic illustration of three example trials with set sizes of 1, 6, and 12 faces, in an angry target block. The target is present in the top and bottom screens, and absent in the middle screen. The screen is visible until the participant makes a response.

The FITC task (Fig. 1) was modelled after Horstmann and Bauland (2006) and used angry/happy photographs from the Pictures of Facial Affect (Ekman & Friesen, 1975), modified to ensure equal recognisability and emotional arousal as described in Schmidt-Daffy (2011). As in a previous study (Horstmann & Bauland, 2006), photographs from one actor (MF) were used.

On each trial, participants had to indicate whether a target face (angry or happy) was present in an array of 1, 6, or 12 faces. On half of the trials, exactly one of these faces showed the target expression on the remaining trials (present trials), and none of the faces showed the target expression (absent trials). This means the task is to detect a target expression in a crowd of faces with the opposite expression, all with the same face identity. Each face was presented with a visual angle of 1.05° (width) × 1.43° (height). Possible stimulus locations were based on an (invisible) 4 (horizontal) × 3 (vertical) array, in which locations had a horizontal distance of 1.86° and a vertical distance of 1.43° from each other. On each presentation, 1, 6, or 12 locations were randomly chosen from this array. Target location was randomly assigned to one of these positions. Actual locations then slightly deviated from the array by randomly adding either −.14°, 0°, or .14° to the array location both in horizontal and in vertical direction. Faces were presented such that their centres corresponded to the resulting locations. The maximum screen area spanned by the array was 6.89° (width) × 4.57° (height).

We presented 300 trials in two blocks, separated by a short break. Participants made a two-alternative forced choice whether the target was present or absent, using the computer keyboard. Target emotion was angry for one block and happy for the other. Block order was randomised across healthy participants; AM started with happy target and BG with angry target. Thus, simple order effects would not result in a group difference between patients and control participants. The target face was shown on its own once before each block, but it was not verbally described. Participants were not asked to verbally describe the facial expression at any stage of the experiment. After presenting the target face, 20 practise trials with feedback followed which were not analysed, and then the experimental trials of the block started. Feedback was given only during practise trials. Each trial started with a 1100 msec fixation cross, followed by the face display which was on until the participant made a response. After the response, the next trial started immediately.

### 2.4 Analysis

We extracted RTs from all correct responses with RT > 200 msec and RT < 3000 msec to calculate mean RTs for each condition; this excluded 15% (AM), 11% (BG), and 5% (0–25%, control group) of trials from analysis. Error rates were computed from all trials. In a signal detection framework, we computed criterion and sensitivity (d′). Search slopes were computed for each individual and each combination of target emotion/target presence by linearly regressing all RTs on set size. We used ANOVA models in SPSS to analyse the control group, and to locate differences between patients and the control group. Because unequal variance in different cells within the control population in an ANOVA design can increase type I error rates (Crawford & Garthwaite, 2007; Crawford, Garthwaite, & Howell, 2009), we confirmed group differences and 2 × 2 interactions using a single-case Bayesian approach as implemented in Crawford's software. Non-significant findings do not require confirmation. Note that for interactions involving a higher order or higher number of levels, no appropriate single-case Bayesian methods are available.

## 3 Results

**Fig. 2:**
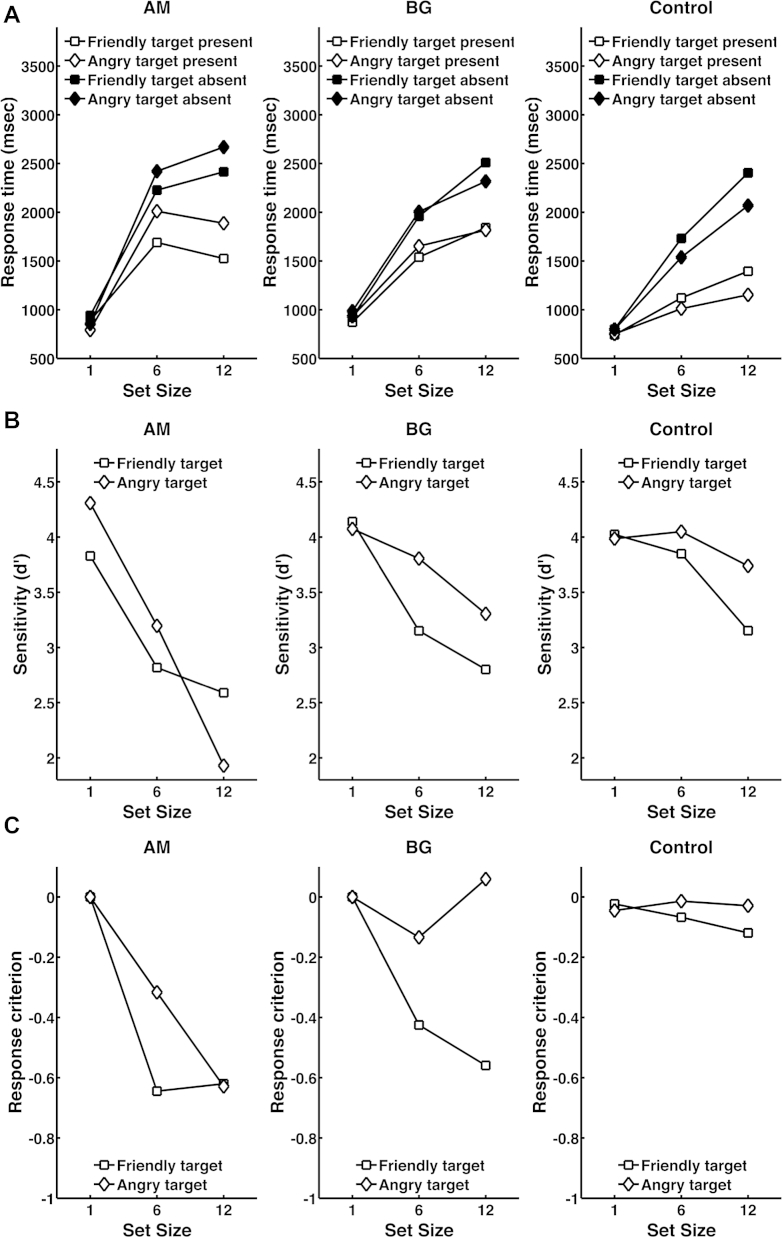
**A** – Response times for AM, BG, and the control group. Both patients responded slower to angry than to happy faces, while healthy individuals show the reverse pattern, termed anger superiority effect. Response times linearly depend on set size in the control group, but are non-linearly dependent on set size in patients. **B** – Sensitivity (d′) for AM, BG, and the control group. **C** – Response criterion for AM, BG, and the control group. Higher values denote a higher probability of reporting an absent target as present, and lower values a higher probability of reporting a present target as absent.

In our control sample, set size, target emotion, and target presence influenced RT as shown previously (see Fig. 2A and Table 1), with a linear impact of set size. This result was confirmed by fitting a linear regression model to predict RT from set size, separately for each combination of target presence and target emotion. An ANOVA on search slope estimates (Table 2) underlines that search slope is influenced by target face – angry target faces have a shallower search slope – and by target presence. There were no effects in an ANOVA on intercepts of the regression model, as expected.

Next, we compared the two patients with the control sample (Fig. 2A, Table 1). Patients responded faster to happy than to angry targets, while healthy individuals showed the opposite pattern, in particular for larger set size (interaction Group × Set size × Emotion). This result was confirmed by comparing patients' search slopes with the control sample which revealed a significant Group × Emotion interaction. On a single individual basis, Bayesian dissociation analysis revealed a significant Group × Emotion interaction for AM (*p* = .017) but not for BG.

Further, patients showed slower RT and steeper search slopes overall. This was confirmed only as a trend in a single-case Bayes approach (one-tailed tests; RTs: AM, *p* < .05; BG, *p* < .10; search slopes: AM, *p* < .05; BG, *p* < .10). Patients also differed from the control group in a stronger non-linear effect of set size (quadratic interaction group × set size: *F*(1, 16) = 18.3; *p* < .005, *η*^2^ = .533) – RTs for the medium set size were disproportionately large.

Reversal of the anger superiority effect in the patients' RTs and search slopes might be due to a different strategy in a speed-accuracy trade-off. In this case, AM and possibly BG should show increased accuracy for angry as opposed to happy targets. Hence, we analysed errors using a signal detection analysis on sensitivity (d′) and response criterion for each combination of set size and target emotion (Table 2, Fig. 2B and C). Impairments in visual search were evident in patients, that is, both sensitivity and criterion depended on set size, unlike in healthy individuals. Importantly, there was no advantage in detection accuracy for angry targets. On the contrary, while both patients and healthy individuals were more sensitive to detect angry than happy faces, this advantage was descriptively less pronounced in patients. To summarise, there is no evidence that a reversal of an anger superiority effect in RT reflects a speed-accuracy trade-off.

## 4 Discussion

Three main findings emerge from our study of two individuals with bilateral and almost complete amygdala lesions in an FITC task with angry and happy face stimuli. First, in patients we observed a reversal of the anger superiority effect seen in healthy individuals. Patients with amygdala lesions were slower to detect an angry target than a happy target, while healthy individuals were faster to detect an angry target. Secondly, this phenomenon was not due to greater response accuracy for the angry targets. Third, patients showed more general impairments in this visual search task, including a trend-level reduction in search speed, and a disproportionately long search time for the medium set size. The latter indicates that they might apply a different search strategy, i.e., searching some empty positions in the array as well.

In summary, our findings suggest that the human amygdala is necessary for prioritising threat information, in keeping with extant theories on amygdala function (LeDoux, 2000) derived from non-human animal research. This view is supported by a previous finding that one of the two individuals reported here, BG, shows reduced startle potentiation by threat-related scene pictures (Becker et al., 2012). It remains the case that another patient with amygdala lesion, SM, is not impaired in prioritising fearful faces under continuous flash suppression (Tsuchiya et al., 2009) – but fearful faces do not necessarily constitute threat signals.

Beyond threat detection, neuroimaging research on human amygdala has proposed relevance detection (Sander, Grafman, & Zalla, 2003) and assessment of subjective arousal (Lewis, Critchley, Rotshtein, & Dolan, 2007; Winston, Gottfried, Kilner, & Dolan, 2005) as a key functions of this structure. Threat detection might be subsumed as a special case of both relevance and arousal assessment. However, in contrast to an impairment in threat detection observed in the present study, the two patients reported here were not impaired in memory advantage for arousing words under capacity limits in a previous report (Bach et al., 2011) although patient SP with broad temporal lobe damage was Anderson and Phelps (2001). Also, patients with surgical unilateral amygdala lesions were not impaired in prioritisation of generally aversive and erotic imagery (Piech et al., 2011) or spider pictures (which are not generally threatening to non-phobic individuals) (Piech et al., 2010). Hence, it appears that general relevance detection and threat detection can be impaired independently from one another. It is possible that they are subserved by different amygdala substructures (some of which might still be functional in these patients), or that one function can be compensated for by other brain circuits while the other function cannot. The latter possibility would account for the apparent differences between neuroimaging and lesion studies.

A previous literature has addressed the amygdala's role in social judgement and explicit, verbal emotion recognition. Lesion studies have shown an impairment in explicit recognition of both angry and fearful faces (Adolphs et al., 1994; Becker et al., 2012) but not in detection of emotions in prosody (Adolphs & Tranel, 1999; Bach et al., 2013), and this could mean that explicit evaluation of facial expression is another function of the amygdala, possibly independent from a function in prioritising threat information.

In line with a previous study (Horstmann & Bauland, 2006), we used only one face identity to reduce variance in dependent measures. To exclude a potential impact of low-level visual features peculiar to this face identity, further work with other face identities is desirable. Also, the fact that we investigated only two individuals with rare selective amygdala lesions renders any generalisation speculative, and similar findings in more individuals are needed to support our conclusions.

In summary, we demonstrate reversal of the anger superiority during visual search in two individuals with amygdala lesion, providing evidence that the human amygdala is involved in rapid detection of threat in faces. This reconciles human and animal lesion literature and confirms the role of this structure for implicit threat processing.

## Financial disclosures

The authors state no conflicts of interest.

## Figures and Tables

**Table 1:** Analysis of raw RTs. Set size (1, 6, 12) × Target emotion (angry, happy) × Target presence (×Group) ANOVA. *p*-values are given after Greenhouse-Geisser correction of degrees of freedom for violations of multisphericity. Effect sizes are reported as *η*^2^.

Effect	df	*ε*	*F*	*η*^2^	*p*
**Results within the control group**					
Set size	2, 30	.909	527.0	.972	<.0005
Emotion	1, 15	1	13.9	.481	=.002
Presence	1, 15	1	335.3	.957	<.0005
Set size × Emotion	2, 30	.631	20.6	.578	<.0005
Set size × Presence	2, 30	.837	229.1	.939	<.0005
Emotion × Presence	1, 15	1	4.4	.227	=.05
Set size × Emotion × Presence	2, 30	.767	<1	.056	n.s.

**Comparison of patient group and control group (effects not involving group are omitted)**					
Group	1, 16	–	6.0	.272	<.05
Group × Set size	2, 32	.643	11.8	.424	<.0005
Group × Emotion	1, 16	1	4.0	.199	=.06
Group × Presence	1, 16	1	2.3	.127	n.s.
Group × Set size × Emotion	2, 32	.629	4.7	.225	<.05
Group × Set size × Presence	2, 32	.829	2.1	.115	n.s.
Group × Emotion × Presence	1, 16	1	<1	.015	n.s.
Group × Set size × Emotion × Presence	2, 32	.769	<1	.003	n.s.

**Table 2:** Analysis of estimated search slopes [Target emotion (angry, happy) × Target presence (×Group) ANOVA], sensitivity (d′), [set size (1, 6, 12) × Target emotion (angry, happy) (×Group) ANOVA], and response criterion [set size (1, 6, 12) × Target emotion (angry, happy) (×Group) ANOVA]. *p*-values are given after Greenhouse-Geisser correction of degrees of freedom for violations of multisphericity. Effect sizes are reported as *η*^2^.

Effect	df	*ε*	*F*	*η*^2^	*p*
**Estimated search slope**					
Results within the control group					
Emotion	1, 15	1	14.5	.461	<.001
Presence	1, 15	1	309.5	.948	<.0005
Emotion × Presence	1, 15	1	<1	.079	n.s.

Comparison of patient group and control group (effects not involving group are omitted)					
Group	1, 16	–	5.2	.244	<.05
Group × Emotion	1, 16	1	4.5	.219	<.05
Group × Presence	1, 16	1	2.9	.152	n.s.
Group × Emotion × Presence	1, 16	1	<1	.005	n.s.

**Sensitivity (d′)**					
Results within the control group					
Set size	2, 30	.591	10.8	.419	<.005
Emotion	1, 15	1	14.9	.498	<.005
Set size × Emotion	2, 30	.686	5.3	.260	<.05

Comparison of patient group and control group (effects not involving group are omitted)					
Group	1, 16	–	7.0	.305	<.05
Group × Set size	2, 32	.595	3.0	.159	=.09
Group × Emotion	1, 16	1	<1	.002	n.s.
Group × Set size × Emotion	2, 32	.675	1.7	.098	n.s.

**Response criterion**					
Results within the control group					
Set size	2, 30	.844	<1	.050	n.s.
Emotion	1, 15	1	2.5	.142	n.s.
Set size × Emotion	2, 30	.690	3.1	.171	=.08

Comparison of patient group and control group (effects not involving group are omitted)					
Group	1, 16	–	22.3	.752	<.0005
Group × Set size	2, 32	.829	8.9	.358	<.005
Group × Emotion	1, 16	1	4.3	.212	=.05
Group × Set size × Emotion	2, 32	.736	1.4	.079	n.s.
